# Optimization of extracellular matrix for primary human hepatocyte cultures using mixed collagen-Matrigel matrices

**DOI:** 10.17179/excli2022-5459

**Published:** 2023-01-04

**Authors:** Lena Seidemann, Sarah Prinz, Jan-Constantin Scherbel, Christina Götz, Daniel Seehofer, Georg Damm

**Affiliations:** 1 Department of Hepatobiliary Surgery and Visceral Transplantation, University Hospital, Leipzig University, Liebigstr. 20, 04103 Leipzig, Germany; 2 Saxonian Incubator for Clinical Translation (SIKT), Leipzig University, Philipp-Rosenthal-Str. 55, 04103 Leipzig, Germany

**Keywords:** in vitro liver models, primary human hepatocytes, extracellular matrix, collagen, Matrigel, sandwich culture

## Abstract

Loss of differentiation of primary human hepatocytes (PHHs) *ex vivo* is a known problem of *in vitro* liver models. Culture optimizations using collagen type I and Matrigel reduce the dedifferentiation process but are not able to prevent it. While neither of these extracellular matrices (ECMs) on their own correspond to the authentic hepatic ECM, a combination of them could more closely resemble the *in vivo* situation. Our study aimed to systematically analyze the influence of mixed matrices composed of collagen type I and Matrigel on the maintenance and reestablishment of hepatic functions. Therefore, PHHs were cultured on mixed collagen-Matrigel matrices in monolayer and sandwich cultures and viability, metabolic capacity, differentiation markers, cellular arrangement and the cells' ability to repolarize and form functional bile canaliculi were assessed by reverse transcription quantitative real-time polymerase chain reaction (RT-qPCR), functional assays and immunofluorescence microscopy. Our results show that mixed matrices were superior to pure matrices in maintaining metabolic capacity and hepatic differentiation. In contrast, Matrigel supplementation can impair the development of a proper hepatocytic polarization. Our systematic study helps to compose an optimized ECM to maintain and reestablish hepatic differentiation on cellular and multicellular levels in human liver models.

## Introduction

A major drawback in primary human hepatocyte (PHH) culture is their dedifferentiation *in vitro* (Elaut et al., 2006[10]). Already in the first few days of cultivation, a reduction of hepatic functions can be observed, e.g. production of plasma proteins, drug metabolizing enzyme activities, glucose metabolism and urea synthesis (Godoy et al., 2013[15]). Intense research over the last decades has tried to overcome this progressive loss of liver-specific functionality. The development of hepatocyte sandwich cultures (SCs), co-cultures with non-parenchymal cells and even more advanced 3-dimensional culturing techniques has revealed the importance of cell-cell and cell-matrix interactions for improving hepatic functions in liver models (Bachmann et al., 2015[2]; Duval et al., 2017[9]; Wei et al., 2018[45]). 

Hepatocytes are epithelial cells that feature a unique type of polarization (Müsch, 2014[29]). Their cell membrane is compartmentalized in specific domains, thus separating blood from bile flow (Pradhan-Sundd and Monga, 2019[32]). The hepatocyte's basal membrane faces the sinusoid. It expresses several transmembrane transporters that manage the exchange of substances with the sinusoidal blood stream (Godoy et al., 2013[15]). Organic anion transporting polypeptide 1B1 (OATP1B1) is a liver-specific transporter of bile acids, hormones and other substances that is expressed uniquely at the basolateral membrane (Obaidat et al., 2012[30]). Adjacent hepatocytes form small apical membrane domains that border the lumen of a bile canaliculus (Slim et al., 2014[38]). The apical membrane is equipped with transport proteins that eliminate bile acids and xenobiotics from the cell, like multidrug resistance-associated protein 2 (MRP2) (Godoy et al., 2013[15]; Ito et al., 2005[20]). Tight junctions (TJs) in the lateral membranes between two neighboring cells flank the apical membrane domains and seal the bile canalicular lumen (Slim et al., 2014[38]). The TJ proteins zonula occludens (ZO)-1 and -2 mediate the connection to the actin cytoskeleton and are crucial elements of the blood-bile barrier (Itoh et al., 2021[21]). Cell-cell contact promoting adherens junctions are situated in the lateral membrane below the TJs and are linked to the actin cytoskeleton via E-cadherin and catenins (Pradhan-Sundd and Monga, 2019[32]). Culturing hepatocytes in a sandwich configuration between two layers of extracellular matrix (ECM) allows them to regain polarity and permits functional analysis of hepatocyte biliary excretion (Deharde et al., 2016[8]; Fardel et al., 2019[11]). 

While cell-cell interactions were sufficiently taken into account with the introduction of sandwich and spheroid cultures, ECM optimization is still insufficiently addressed. Only 16-22 % of the liver volume is composed of ECM (Ye et al., 2019[48]). Although scarcely distributed, the ECM is an integral part of the complex liver architecture and the restoration of cell-ECM contacts is crucial for the preservation of the differentiated hepatic phenotype in cell culture (Fraczek et al., 2013[12]; Ye et al., 2019[48]). *In vivo*, the distribution of ECM components varies between the different parts of the liver acinus, the smallest organizational unit of the liver (Godoy et al., 2013[15]; Martinez-Hernandez and Amenta, 1993[26]). Collagen type I is present all over the liver lobule, while the formation of a basement membrane (BM) is only observed in the portal and central spaces. However, collagen type IV, a typical BM element, can also be found in the space of Disse (Martinez-Hernandez and Amenta, 1993[26]). The most commonly applied ECM components for hepatocyte cultures are collagen type I and Matrigel, a commercially available BM matrix extract from Engelbreth-Holm-Swarm (EHS) mouse sarcoma (Serna-Márquez et al., 2020[35]; Zeilinger et al., 2016[49]). Matrigel is a mixture of the BM proteins collagen type IV, laminin, entactin and heparan sulphate proteoglycane (Benton et al., 2014[4]; Hughes et al., 2010[19]). Culturing hepatocytes on either collagen type I or Matrigel alone, does not sufficiently reflect the ECM composition of the liver (Benton et al., 2014[4]; Hughes et al., 2010[19]; Martinez-Hernandez and Amenta, 1993[26]). A combination of these two ECM components, however, could result in a closer approximation to the *in vivo* situation. Attempts to mimic an *in vivo*-like ECM have been made by utilizing decellularized liver ECMs with diverging results (Alevra Sarika et al., 2020[1]; Bual and Ijima, 2019[7]). Serna-Márquez et al. additionally examined the influence of different combinations of collagen type I and Matrigel conjugated to polyacrylamide hydrogels and reported a positive influence of collagen on viability and aggregation of rat hepatocytes cultured on soft conditions (Serna-Márquez et al., 2020[35]). In a previous study, we observed that the application of Matrigel in combination with the classic collagen culture can have a beneficial influence on hepatocyte morphology and polarization (Deharde et al., 2016[8]). To the best of our knowledge, mixed collagen-Matrigel matrices have not been evaluated as ECM for primary hepatocyte cultures of human origin before.

The aim of this study was to systematically analyze the influence of varying mixing ratios of collagen type I and Matrigel on hepatocyte differentiation and morphology. First, PHHs were cultured in monolayer cultures (MCs) on different collagen coatings (C), supplemented with varying percentages of Matrigel (M). Cell viability, metabolic activity and hepatic differentiation markers were analyzed by reverse transcription quantitative real-time polymerase chain reaction (RT-qPCR) and functional assays over the course of 6 days. For further morphological examination, PHHs were cultured in SCs. Hepatocyte morphology, polarization and the formation of bile canaliculi were evaluated by fluorescence microscopy. Our data reveal that supplementing collagen type I-based ECMs with Matrigel supports the maintenance of hepatocyte differentiation and their metabolic capacity in MCs. In SCs, however, Matrigel can impair the development of a proper hepatocytic polarization. Our systematic study revealed how to compose an optimized ECM to maintain and reestablish hepatic differentiation on cellular and multicellular levels.

## Materials and Methods

### Isolation of primary human hepatocytes

Macroscopically tumor-free liver tissue samples were obtained from patients undergoing liver surgery at Leipzig University Hospital. All patients gave their informed consent according to the ethical guidelines of the Medical Faculty of Leipzig University (Ethical vote: registration number 322/17-ek, date 2020/06/10 ratified on 2021/11/30 and registration number 006/17-ek, date 2017/03/21 ratified on 2019/02/12). PHHs were isolated from the liver tissue samples by a two-step EGTA/collagenase perfusion technique as described previously (Pfeiffer et al., 2015[31]). The resulting cell pellet was washed with phosphate buffered saline (PBS; Gibco, Paisley, UK) and the cells were resuspended in PHH culture medium (William's Medium E with GlutaMAX™ (WME; Gibco), supplemented with 10 % fetal calf serum (FCS; Merck Biochrom, Berlin, Germany), 15 mM HEPES, 1 % nonessential amino acids (MEM NEAA 100x), 1 mM sodium pyruvate, 100 U/100 μM penicillin/streptomycin (all provided by Gibco), 40 U/ml insulin (Sanofi Aventis, Frankfurt am Main, Germany) and 1 μg/ml dexamethasone (JENAPHARM, Jena, Germany)). Afterwards, the cell number and viability were determined in a Neubauer counting chamber by Trypan blue staining (Sigma-Aldrich, St. Louis, MO, USA).

### Hepatocyte monolayer culture

For the MCs, cell culture plates were previously coated with ECMs based on collagen type I (C), supplemented with ascending percentages of phenol red-free Matrigel (M; Corning Inc., Corning, NY, USA). The type I collagen solution was previously prepared from rat tail in our own laboratory according to the protocol of Rajan et al. (2006[33]). Collagen and Matrigel were diluted with PBS to 0.076 μg/μl working solutions, which were mixed in the different ratios stated in Table 1(Tab. 1). A volume of 50 μl of each final solution was used for coating 96-well culture plates and 250 μl for 24-well culture plates (both from Greiner Bio-One GmbH, Frickenhausen, Germany). All steps were conducted on ice and with pre-cooled pipette tips. Then, the plates were incubated for 1 h at RT to allow a polymerization of the coatings. PHHs were seeded on top of the gels at a density of 300,000 viable cells/cm^2^ in 96-well plates and 200,000 viable cells/cm^2^ in 24-well plates. After an initial adherence phase of 4 h at 37 °C, 5 % CO_2_ in a humidified incubator (Heraeus HERA-Cell240, Kendro Laboratory Products GmbH, Langenselbold, Germany), non-attached cells were removed by a washing step with PBS, followed by further adherence overnight in PHH culture medium at 37 °C, 5 % CO_2_. The cell culture medium was changed the next morning and then refreshed every other day for up to 6 days. The cell culture supernatants were stored at -80 °C for later analyses of enzyme activities and hepatic metabolites. The time until supernatant collection on day 0 was 8 to 14 h. Time until supernatant collection on the later time points was always 48 h. Therefore, the values of read-outs from enzyme activity and metabolite assays were normalized to the time until supernatant collection (and protein content).

### Hepatocyte sandwich culture

For the SCs, PHHs were cultured in 8-well slides (ibidi GmbH, Gräfelfing, Germany) between two layers of pure collagen type I (100C), pure Matrigel (100M) or a mixture of 90 % collagen and 10 % Matrigel (90C/10M). Slides were coated with 40 µl/cm^2^ poly-l-lysine (Sigma-Aldrich) in advance. Matrigel was diluted with PHH culture medium to 4 µg/µl. Collagen was diluted with WME 10x (Sigma-Aldrich) to 3 µg/µl. The pH value was adjusted to 7.4 by careful addition of NaOH. For 100C and 90C/10M coatings, 55 µl of the respective final solutions were administered per well. For 100M coatings, 100 µl were applied. All steps were conducted on ice and with pre-cooled pipette tips. Then, slides were incubated for 1 h at 37 °C, 5 % CO_2_ in a humidified incubator to allow polymerization of the gels. PHHs were seeded on top of the gels at a density of 200,000 viable cells/cm^2^. After an initial adherence phase of 4 h at 37 °C, 5 % CO_2_ in a humidified incubator, non-attached cells were removed by a washing step with PBS. A gel overlay equal in volume and concentration to the bottom layer was added on top of the cells. After 1 h at 37 °C, 5 % CO_2_ in a humidified incubator, the top layer was polymerized and the SCs were incubated in PHH culture medium at 37 °C, 5 % CO_2_ in a humidified incubator for up to 6 days. The cell culture medium was refreshed every other day.

### XTT assay

Cell activity was determined as a function of redox potential with the colorimetric XTT assay (Cell Proliferation Kit II (XTT; Hoffmann-La Roche AG, Basel, Switzerland). The assay is based on the reduction of the yellow tetrazolium salt XTT (2,3-bis-(2-methoxy-4-nitro-5-sulfophenyl)-2H-tetrazolium-5-carboxanilid) by dehydrogenase enzymes in the mitochondria of metabolically active cells, resulting in orange formazan. The assay was performed on MCs from 5 donors according to the manufacturer's protocol. Absorbance was measured after 4 h incubation at wavelengths of 492 and 690 nm with a microplate reader (Synergy H1, Biotek, Bad Friedrichshall, Germany). According to the manufacturer's protocol, absorbance values detected at 690 nm were then subtracted from the values measured at 492 nm to exclude falsification by background artefacts. All measurements were performed with 6 technical replicates.

### Bicinchoninic Acid (BCA) assay

Protein concentrations were determined using the bicinchoninic acid (BCA) assay. Cell lysis buffer (500 ml Dulbecco's phosphate buffered saline (DPBS, Gibco), 5 ml 10 % sodium dodecyl sulfate (SDS), 2.5 ml Triton X-100, Trizma HCl 3. 94 g (all from Sigma-Aldrich) was added to each well of the MC from 5 donors. Subsequently, the cell culture plates were stored at least overnight at -20 °C before thawing. The resulting solution was transferred to reaction vials. After ultrasonic treatment, the soluble cellular components were separated by centrifugation (10,000xg, 10 min) and the supernatants were diluted 1:2. 20 µl of each sample were transferred to a 96-well plate in triplicate. A standard curve based on serial diluted concentrations of bovine serum albumin (BSA; Sig-ma-Aldrich) with an initial concentration of 2 mg/ml was prepared. Finally, 300 µl of BCA working solution was added to each well, which was prepared from BCA reagents A and CuSO_4_ (both from Sigma-Aldrich) at a ratio of 1:51. Plates were incubated for 30 min at 37 °C in the dark and absorbance was measured at 550 nm with a microplate reader (Synergy H1, Biotek). All measurements were performed with 3 technical replicates.

### Analysis of enzyme activities

Lactate dehydrogenase (LDH), alanine aminotransferase (ALT), aspartate aminotransferase (AST) and γ-glutamyltranspeptidase (g-GT) are intracellular hepatic enzymes that are released upon damage of the cell membrane. The enzymatic activity of LDH, ALT, AST and g-GT was measured in the cell culture supernatants of MCs from 5 donors, which were grown as duplicates for each ECM condition. Supernatants of each duplicate were pooled and analyzed using Dialab reaction kits (Dialab GmbH, Sasbach, Germany). All assays were performed according to the manufacturer's protocol. Absorbances of ALT, AST and LDH assays were measured at 340 nm and the absorbance of the g-GT assay at 405 nm with a microplate reader (Synergy H1, Biotek). All measurements were performed with 3 technical replicates.

### Quantification of hepatic metabolites

The metabolic activity of hepatocytes can be assessed by measuring the amount of urea, lactate and glucose released into the cell culture supernatant. The metabolite concentrations of MC cell culture supernatants from 5 donors, pooled from duplicates of each ECM condition, were measured with Dialab reaction kits following the manufacturer's instructions. Absorbances from lactate and urea assays were measured at 340 nm and of the glucose assay at 405 nm with a microplate reader (Synergy H1, Biotek). All measurements were performed with 3 technical replicates.

### RT-qPCR analyses

For the determination of messenger RNA (mRNA) expression levels of CK-18 (keratin 18), E-Cad (E-cadherin), ALB (albumin), FGA (fibrinogen alpha chain), FGB (fibrinogen beta chain), FGG (fibrinogen gamma chain), HNF4A (hepatocyte nuclear factor 4 alpha), CYP3A4 (cytochrome P450 family 3 subfamily A member 4) and CYP2C9 (cytochrome P450 family 2 subfamily C member 9), RT-qPCR was performed on MCs from 3 donors. Total RNA from 2 independent wells of each donor was isolated by Trizol® (Invitrogen, Karlsruhe, Germany) extraction according to manufacturer's protocol. RNA concentrations were determined with a spectrophotometer (NanoDrop 2000, Thermo Fisher, Osterode, Germany). Reverse transcription was conducted with the Quanti-Nova Reverse Transcription Kit (Qiagen N.V., Venlo, Netherlands) in an Applied Biosystems ProFlex™ cycler (Applied Biosystems, Waltham, MA, USA) following the manufacturer's instructions. Total RNA quantities of 1 µg were applied per reaction. The thermocycling profile is shown in detail in Table 2(Tab. 2).

For the qPCR reactions, the Applied Biosystems 7500 Real Time PCR System (Thermo Fisher Scientific Inc., Waltham, MA, US) was utilized with a cDNA quantity of 40 ng applied for each reaction. Specific primers for ALB, CK-18, CYP2C9, EEF2 (Eukaryotic translation elongation factor 2), FGA, FGB and FGG were designed with NCBI/Primer-BLAST and Primer3web version 4.1.0 softwares and purchased from biomers.net GmbH (Ulm, Germany). Specific primers for 18S_rRNA (18S ribosomal RNA), CYP3A4, E-Cad, GUSB (Glucuronidase beta) and HNF4A and QuantiNova SYBR Green PCR Master Mix were obtained from Qiagen N.V. Primer efficiencies were calculated from slopes of cDNA serial dilution Cq values. Primer specificity was confirmed by melt curve analyses and gel electrophoresis. Primer details are listed in Table 3(Tab. 3). The cycling profile is detailed in Table 4(Tab. 4). All samples were measured in triplicates. EEF2, 18S_rRNA and GUSB served as reference genes. Relative gene expression levels were calculated by normalization to the day 0 gene expression level and the geometric average of the three reference genes as described in Vandesompele et al. (2002[42]).

### Dead or alive assay

An intact plasma membrane and intracellular esterase activity are characteristic features of living cells. Cellular integrity of the sandwich-cultured hepatocytes from 3 donors was investigated by a dead or alive assay over the course of six days. For this purpose, cells were washed twice with PBS, followed by 30 min of incubation with 250 µl per well of a prepared solution containing 5 μM calcein acetoxymethyl ester (calcein AM; AnaSpec Inc., Seraing, Belgium) and 2 μM ethidium homodimer-1 (Promokine, Heidelberg, Germany) dissolved in phenolred-free RPMI (Biochrom GmbH, Berlin, Germany). Calcein AM is a non-fluorescent substance, which is converted to the green fluorescent calcein by intracellular esterases of viable cells. Ethidium homodimer-1 binds to the DNA of cells with damaged plasma membranes and leads to red fluorescence marking of their nuclei. Images were taken with a Keyence microscope BZ-9000 (Keyence GmbH, Neu-Isenburg, Germany). For each time point and culture condition, dead and alive cells were counted manually in 3 randomly chosen microscopy images.

### Immunofluorescence staining

Hepatocyte polarization was investigated by immunofluorescence staining of antigens specifically expressed in the distinct hepatocellular membrane domains. Sandwich-cultured hepatocytes from 3 donors were washed with PBS and fixed with 4 % formaldehyde (Carl Roth GmbH + Co. KG, Karlsruhe, Germany) for 20 min. After washing three times with PBS, cells were permeabilized with 0.2 % Triton X-100 for 20 min. After a further washing step, unspecific binding sites were blocked with 2.0 % BSA for 1 h. Then cells were incubated with primary antibodies (see Table 5(Tab. 5)) over night at 4 °C. Cells incubated without primary antibodies served as negative controls. The immunolabeled cells were washed three times with PBS and incubated with the secondary antibodies (see Table 5(Tab. 5)) for 1 h. Furthermore, cell nuclei were stained with Hoechst 33342 (Thermo Scientific, Rockford, IL, USA) for 15 min and the actin filaments of the cytoskeleton with phalloidin 555 (Abcam, Cambridge, England) for 20 min. Images were taken with a confocal microscope (LSM 700, Carl Zeiss GmbH, Göttingen, Germany).

### CDF assay

The formation of bile canaliculi by PHHs in SCs from 3 donors was visualized microscopically with the biliary excreted fluorescent CDF (5- (and 6) -carboxy-2-, 7-dichlorfluorescein). The compound 5- (and 6) -carboxy-2-, 7-dichlorofluorescein-diacetate (CDF-DA, Santa Cruz Biotechnology Inc., Dallas, TX, US) diffuses passively into cells, where it is hydrolyzed by intracellular esterases into the fluorescent product 5- (and 6) -carboxy-2-, 7-dichlorfluorescein (CDF). The transport protein MRP2 (multidrug resistance-associated protein 2) which is situated in the apical hepatocellular membrane, transports CDF into the bile. The experiment was performed according to the method established by Bi et al. (2006[5]), with some modifications as reported in Deharde et al. (2016[8]). Subsequently, images were taken with a fluorescence microscope (Keyence BZ 9000, Keyence GmbH) after a period of 15-20 min.

### Statistical analyses

Our study was performed as explorative analysis with the goal of testing a broad set of different matrices. Therefore, high amounts of cells were needed, restricting the study design to a small set of donors due to economic reasons. To strengthen conclusions, we perfomed assays on multiple levels to validate our results. Due to the explorative design of the study we decided for parametric testing despite the small sample size. Statistical analyses and chart design were performed with GraphPad Prism 7 (GraphPad Software, San Diego, CA, USA). Data are expressed as the mean + standard error of mean (SEM). For significance testing a repeated measures two-way ANOVA (with the two factors time and coating) was employed followed by a Bonferroni correction for multiple testing. Statistical significance was assumed at * p ≤ 0.0332, ** p ≤ 0.0021, *** p ≤ 0.0002, **** p < 0.0001. PCR data are shown as the mean of normalized fold gene expressions + SEM on a log10-scale. Statistics were performed on ΔCT values.

## Results

### Long-term metabolic activity of PHHs is higher in monolayer cultures on mixed matrices than on pure collagen type I or Matrigel coatings

The metabolic capacity of PHHs cultured for up to 6 days in monolayers on the various ECM compositions was assessed on several levels: mitochondrial activity, protein con-centration, cell integrity and metabolite production. 

**Figure 1:**
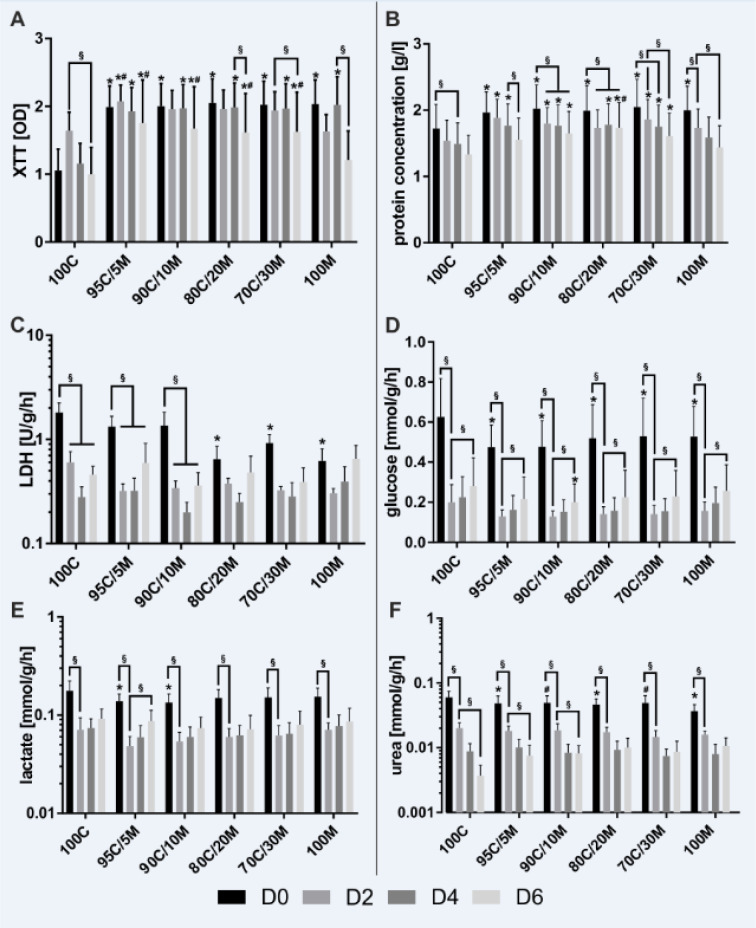
Metabolic activity of primary human hepatocytes (PHHs) in monolayer cultures (MCs) on different extracellular matrices (ECMs). PHHs were cultured for up to 6 days on pure collagen type I (100C), on ECMs based on collagen type I (C), supplemented with ascending percentages of Matrigel (M): 95C/5M, 90C/10M, 80C/20M, 70C/30M or on pure Matrigel (100M). PHHs were investigated for (A) metabolic activity determined by the conversion of XTT (2,3-bis-(2-methoxy-4-nitro-5-sulfophenyl)-2H-tetrazolium-5-carboxanilid); (B) protein concentration measured with the BCA (bicinchoninic acid) assay; (C) enzyme activity of LDH (lactate dehydrogenase) and the production of (D) glucose, (E) lactate and (F) urea. LDH, glucose, lactate and urea were measured in cell culture supernatants and normalized to the protein concentration and duration of cell culture until supernatant collection. Data are shown as the mean + SEM, n = 5, two-way ANOVA and Bonferroni correction for multiple testing, statistical significance was assumed at p ≤ 0.0332. Significant differences between PHHs cultured on one of the various matrices in comparison to PHHs cultured on 100C of the same time point are marked with *. Significant differences between PHHs cultured on one of the various matrices in comparison to PHHs cultured on 100M of the same day are marked with #. Significant differences observed at different time points of PHHs cultured on the same ECM are marked with §. Selected comparisons are shown; for details on the statistical evaluation, see Supplementary Table 1A, supplementary data.

Mitochondrial activity of viable cells was measured with the XTT assay (Figure 1A(Fig. 1)). It revealed a stable mitochondrial activity over the first 4 days of culture and a significant decline on day 6 in pure collagen type I (100C)-cultured cells and in cells cultured on matrices containing 20 % and more Matrigel. In general, PHHs cultured on mixed matrices for 6 days presented a higher mitochondrial activity than the cells on pure collagen type I or Matrigel coatings. Furthermore, PHHs cultured on pure collagen type I showed a reduced cell activity in comparison to hepatocytes kept on almost any other coating. Detection of changes in protein concentrations can serve as marker for cell number stability, but also reflects cellular growth. Alike the pattern seen in the XTT assay results, protein concentrations of PHHs cultured on pure collagen type I were lower than those of PHHs on mixed coatings for most time points (Figure 1B(Fig. 1)). However, they did not differ from the protein concentrations of the PHHs cultured on pure Matrigel on days 2 to 6. In the cells cultured on the mixed ECM containing the highest percentage of Matrigel (70 % collagen type I/30 % Matrigel (70C/30M)) or collagen (95 % collagen type I/5 % Matrigel (95C/5M)) as well as on pure Matrigel (100M) and 100C, a gradual decline in the protein concentrations was observed. Cultures on 90 % collagen type I / 10 % Matrigel (90C/10M) and 80 % collagen type I/20 % Matrigel (80C/20M) showed a decrease in comparison to the initial protein concentration, but stabilized their protein content over days 4 to 6. Release of intracellular lactate dehydrogenase (LDH) is a marker for reduced cell integrity. All cultures showed high initial values of LDH release in comparison to the subsequent course of the cell culture reflecting the remaining cell isolation stress (Figure 1C(Fig. 1)). Supplementation of Matrigel in the coatings seemed to account for less initial cell damage as was reflected by lower LDH activities on day 0 by PHHs cultured with 20 % Matrigel and more. On later days however, LDH release was stable and did not differ from PHH cultures on the other matrices indicating no negative impact of the ECMs on cell viability. The additionally analyzed hepatocyte enzyme release of aspartate aminotransferase (AST), alanine aminotransferase (ALT) and gamma-glutamyl transferase (g-GT) did not reveal any differences in the long-term cultivation on different ECMs (Supplementary Figure 1A-C) and therefore confirm the LDH release results. The hepatic metabolites glucose, lactate and urea that were secreted to the cell culture supernatants display the hepatic synthesis and metabolic capacities (Figure 1D-F(Fig. 1)). For all three metabolites, an initial decline on day 2 was noticed. Glucose increased again on day 4 in all cell cultures. The urea production, however, declined further in the PHHs cultured on ECMs containing higher amounts of collagen type I (100C, 95C/5M and 90C/10M). Summing up, especially the investigations on mitochondrial activity and protein concentrations of PHHs provide indications, that for long-term monolayer cultures, mixed collagen-Matrigel coatings deliver favorable conditions.

### Culturing PHHs in monolayers on mixed matrices promotes stability of differentiation marker mRNA expressions

**Figure 2:**
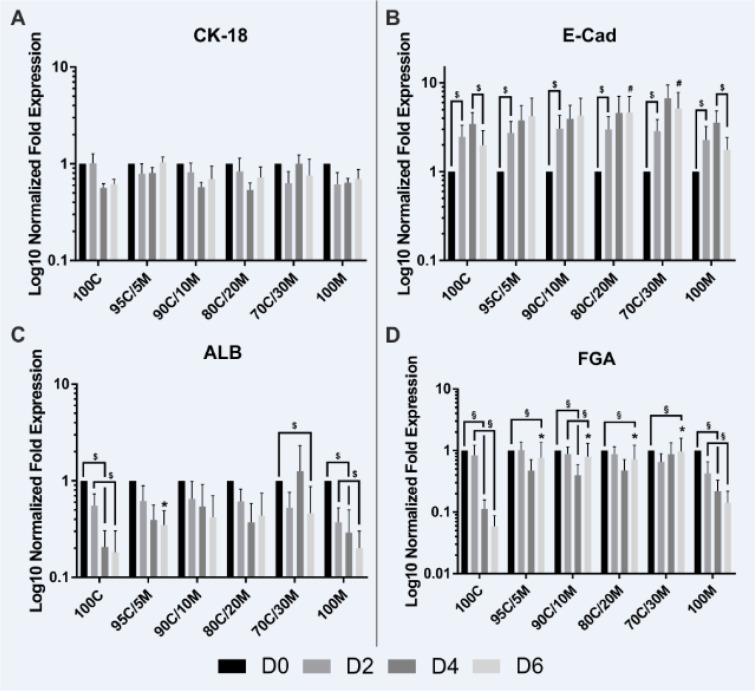
Gene expression of differentiation markers in primary human hepatocytes (PHHs) in monolayer cultures (MCs) on different extracellular matrices (ECMs). PHHs were cultured for up to 6 days on pure collagen type I (100C), on ECMs based on collagen type I (C), supplemented with ascending percentages of Matrigel (M): 95C/5M, 90C/10M, 80C/20M, 70C/30M or on pure Matrigel (100M). Messenger RNA levels of (A) CK-18 (keratin 18), (B) E-Cad (E-cadherin), (C) ALB (albumin) and (D) FGA (fibrinogen alpha chain) were determined by RT-qPCR. Fold gene expressions were normalized to day 0 of each cultivation type. Data are shown as the mean of normalized fold gene expressions + SEM on a log10-scale, n = 3, two-way ANOVA and Bonferroni correction for multiple testing, statistics were performed on ΔCT values, statistical significance was assumed at p ≤ 0.0332. Significant differences between PHHs cultured on one of the various matrices in comparison to PHHs cultured on 100C of the same day are marked with *. Significant differences between PHHs cultured on one of the various matrices in comparison to PHHs cultured on 100M of the same day are marked with #. Significant differences observed at different time points of PHHs cultured on the same ECM are marked with §. Selected comparisons are shown; for details on the statistical evaluation, see Supplementary Table 1B, supplementary data.

To evaluate hepatocyte differentiation marker gene expressions in dependence of the applied ECM compositions, mRNA levels of keratin 18 (CK-18), E-cadherin (E-Cad), albumin (ALB) and fibrinogen alpha chain (FGA) were analyzed by RT-qPCR (Figure 2A-D(Fig. 2)). The typical hepatocyte marker CK-18 was the only differentiation marker that was not affected by culture time or condition. Expression of the epithelial cell-cell adhesion protein E-Cad increased significantly on day 2 of all MCs, but decreased again on day 6 in the 100C- and 100M-cultured PHHs. In contrast, PHHs cultured on the collagen and Matrigel-containing ECMs had a stable E-Cad expression. On day 6, E-Cad expression was significantly higher in the 80C/20M and 70C/30M cultures than in PHHs kept on 100 % Matrigel. Synthesis of the circulating plasma proteins albumin and fibrinogen are key-features of healthy hepatocytes. mRNA expressions of ALB and FGA significantly decreased on days 4 and 6 in PHHs cultured on 100C and 100M. In the mixed ECM cultures, FGA also decreased on day 6, but was still higher than in the 100C cultures. ALB expression was stable in most mixed ECM cultures. Fibrinogen beta and gamma chain (FGB and FGG) mRNA expressions were also analyzed and a negative influence on them was especially observed for the 100M coating that led to a decline already on day 2 (Supplementary Figure 2A-B). Most of the collagen-containing coatings led to a reduced expression of fibrinogens on day 4. Only the 95C/5M-cultured PHHs showed a stable FGB expression. FGG expression was higher on day 6 in the 90C/10M- and 80C/20M-cultured PHHs than in 100C-cultured cells. Thus, PHH MCs on coatings containing both collagen and Matrigel showed a less altered expression profile of hepatocyte differentiation markers than PHHs cultured on either pure collagen type I or Matrigel.

### mRNA expression of HNF4A and CYP2C9 is higher in long-term cultured PHH MCs on collagen-Matrigel mix ECMs than on pure collagen type I or Matrigel

**Figure 3:**
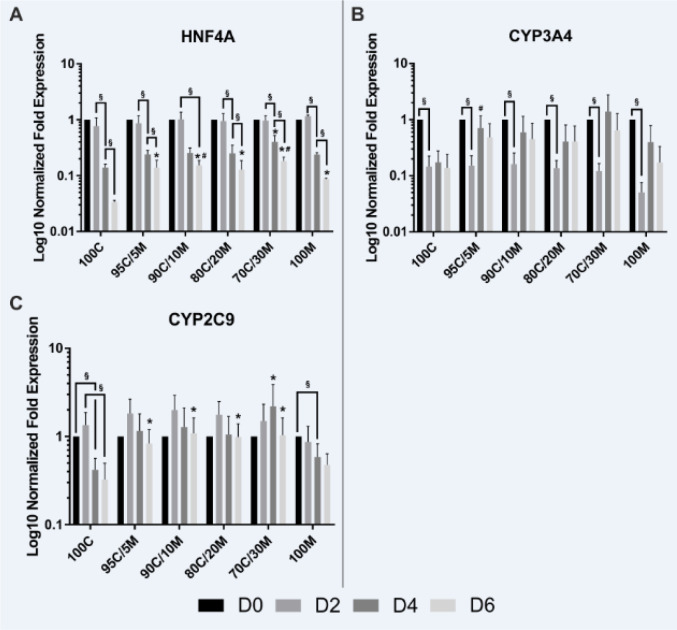
Gene expression of HNF4A and its downstream targets in primary human hepatocytes (PHHs) in monolayer cultures (MCs) on different extracellular matrices (ECMs). PHHs were cultured for up to 6 days on pure collagen type I (100C), on ECMs based on collagen type I (C), supplemented with ascending percentages of Matrigel (M): 95C/5M, 90C/10M, 80C/20M, 70C/30M or on pure Matrigel (100M). Messenger RNA levels of (A) HNF4A (hepatocyte nuclear factor 4 alpha), (B) CYP3A4 (cytochrome P450 family 3 subfamily A member 4) and (C) CYP2C9 (cytochrome P450 family 2 subfamily C member 9) were determined by RT-qPCR. Fold gene expressions were normalized to day 0 of each cultivation type. Data are shown as the mean of normalized fold gene expressions + SEM on a log10-scale, n = 3, two-way ANOVA and Bonferroni correction for multiple testing, statistics were performed on ΔCT values, statistical significance was assumed at p ≤ 0.0332. Significant differences between PHHs cultured on one of the various matrices in comparison to PHHs cultured on 100C of the same day are marked with *. Significant differences between PHHs cultured on one of the various matrices in comparison to PHHs cultured on 100M of the same day are marked with #. Significant differences observed at different time points of PHHs cultured on the same ECM are marked with §. Selected comparisons are shown; for details on the statistical evaluation, see Supplementary Table 1C, supplementary data.

Hepatocyte nuclear factor 4 alpha (HNF4A), the hepatic key metabolic transcription factor and two of its downstream targets that are members of the cytochrome P450 superfamily (CYP3A4 and CYP2C9) (Jover et al., 2001[22]) were chosen as central regulators of hepatocyte metabolism and their mRNA expression levels were analyzed by RT-qPCR (Figure 3A-C(Fig. 3)). The expression of HNF4A declined under all culture conditions over the 6 observed days. But PHHs cultured on mixed matrix coatings still showed a higher HNF4A expression on day 6 than on 100C and partly also than on 100M. The expression levels of CYP3A4 already decreased on day 2 independently of the applied coatings, but remained stable thereafter. The only difference that could be observed between the different cultivation conditions was a higher CYP3A4 expression on day 4 on 95C/5M in comparison to 100M. For CYP2C9, however, only PHHs cultured on pure collagen type I or Matrigel showed a decline in their mRNA expression levels over the course of time. PHHs cultured on mixed matrices presented a stable expression, which was even increased by trend on day 2. Taken together, long-term cultured PHHs showed higher expression levels of HNF4A and CYP2C9 when cultured on mixed matrices than on pure collagen type I and for HNF4A also than on pure Matrigel. Therefore, the PHHs cultured on mixed matrices showed a reduced dedifferentiation. The stabilizing effect of the collagen-Matrigel mix ECM was, however, not as clear as seen for the other differentiation markers.

### Cell viability decreases only in PHH SCs on pure Matrigel

**Figure 4:**
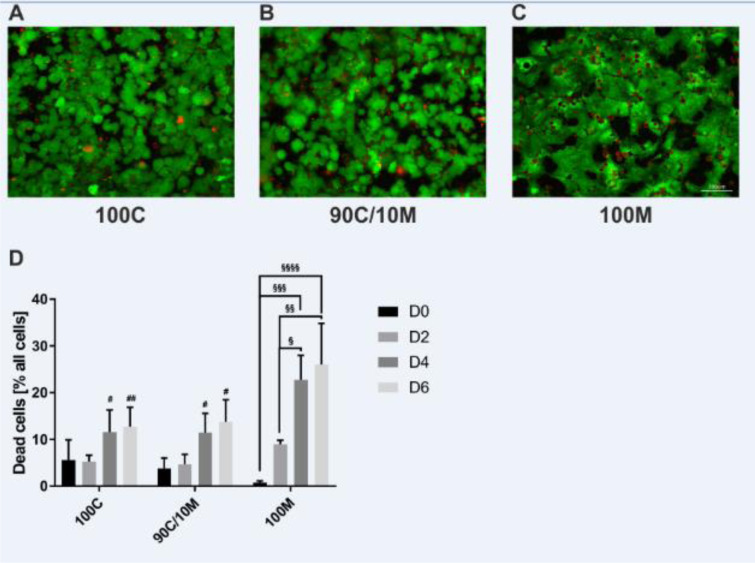
Cell viability of primary human hepatocytes (PHHs) in sandwich cultures (SCs) between different extracellular matrices (ECMs). PHHs from 3 donors were cultured for up to 6 days between two layers of (A) pure collagen type I (100C), (B) collagen type I supplemented with 10 % Matrigel (90C/10M) or (C) pure Matrigel (100M). Viable cells were detected by the intracellularly converted fluorescent calcein (green). Nuclei of dead cells appear red after binding of the fluorescent ethidium homodimer-1. (A-C) Representative fluorescence microscopy images of PHH SCs after 6 days of cultivation are shown. Magnification: 200x. Scale Bar 100 µm. (D) Cell counts were assessed manually in 3 randomly chosen microscopy images per ECM type on days 0, 2, 4 and 6 of cultivation. Data are shown as mean + SEM, n = 3, two-way ANOVA and Bonferroni correction for multiple testing, statistical significance was assumed at * p ≤ 0.0332, ** p ≤ 0.0021, *** p ≤ 0.0002, **** p < 0.0001. Significant differences between PHHs cultured on one of the various matrices in comparison to PHHs cultured on 100M of the same day are marked with #. Significant differences observed at different time points of PHHs cultured on the same ECM are marked with §.

The supplementation of Matrigel showed beneficial effects in MCs. Therefore, we chose the supplementation of 10 % Matrigel for further investigations in SCs as this concentration was situated between positive effects detected when using low Matrigel concentrations and first detrimental effects observed in higher Matrigel concentrations. The influence on cellular arrangement and integrity of the cell layer was investigated for the chosen ECM composition in comparison to pure ECMs. Therefore, cell viability was assessed in the sandwich-cultured PHHs by a fluorescent dead or alive assay (Figure 4A-D(Fig. 4)). Cell counts of dead cells showed an increase over the course of 6 culture days in the 100M cultures. In these cultures, numbers of dead cells were also significantly higher than in the collagen-containing cultures of culture days 4 and 6. Therefore, cell viability was better sustained in the collagen-containing SCs than on pure Matrigel.

### Increasing Matrigel content leads to a loss of hepatocytic polarization in PHH sandwich cultures

For the investigation of the influence of different ECM compositions on hepatocyte morphology and polarity, SCs were conducted with PHHs from 3 different donors for 6 days. The cellular morphology and the multicellular arrangement was investigated using Phalloidin (actin cytoskeleton) and Hoechst (nuclei) staining. In general the actin expression showed a very donor dependent pattern with highest concentrations between adjacent cells. Additionally, the expression correlated with formation of bile canaliculi and surrounded the canalicular network. The multicellular arrangement showed a homogeneous, confluent distribution for 100G and 90C/10M. In contrast, 100M showed additionally to a confluent arrangement also a clustered arrangement forming strains of cells alternating with elliptical voids. 

**Figure 5:**
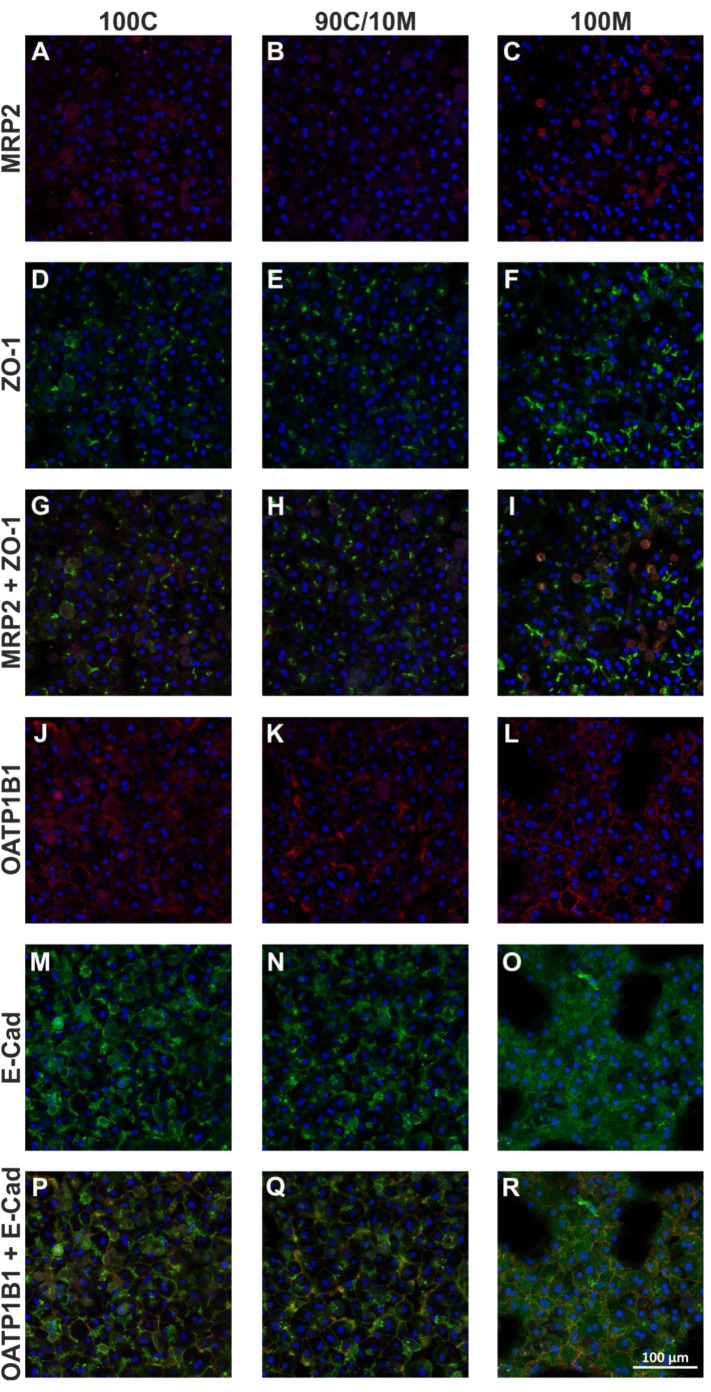
Investigation of primary human hepatocyte (PHH) polarization in sandwich cultures (SCs) between different extracellular matrices (ECMs). PHHs from 3 donors were cultured for 6 days between two layers of pure collagen type I (100C; first column), collagen type I supplemented with 10% Matrigel (90C/10M; second column) or pure Matrigel (100M; third column). The expression of the apical membrane-associated MRP2 (multidrug resistance-associated protein 2; A-C; red) and the tight junction protein ZO-1 (zonula occludens-1; D-F; green) are displayed individually and merged (G-I). Also, the expression of the basal membrane-associated OATP1B1 (organic anion transporting polypeptide 1B1; J-L; red) and the adherens junction-associated protein E-cadherin (M-O; green) are displayed individually and merged (P-R). Cell nuclei were stained with Hoechst (blue). Imaging analysis was performed by three different investigators independently. Representative fluorescence microscopy images are shown. Magnification: 200x. Scale Bar 100 µm. A comprehensive presentation of images from all three donors can be found in Supplementary Figures 3-5, supplementary information. Supplementary Figure 6 shows PHH morphology and multicellular arrangement in SCs between different ECMs.

To analyze the presence of the specific membrane domains of the polarized hepatocyte, immunofluorescence microscopy with antigen-specific antibodies addressing the following targets was conducted: the transmembrane transporter MRP2 that is situated in the apical membrane facing the bile canaliculi; the tight junction protein ZO-1 and the adherens junction-associated protein E-cadherin which are situated in the lateral membrane at cell-cell contacts and the transport protein OATP1B1 that is localized in the basolateral membrane compartment (Figure 5A-R(Fig. 5)). The sinusoid-facing basolateral membrane domain is either located at the border of the cell arrangement or at the top/bottom of the cell layer facing the ECM which is out of focus in micrographs. Consequently, a basolateral staining should either be visible at the border towards bigger voids or as a diffuse staining over the cellular shape.

### PHH SCs between two layers of 100 % collagen

PHHs adhered homogeneously and in high density forming hepatocyte-typical hexagonal shapes. The ZO-1 expression was visible as branched lines between neighboring cells. MRP2 expression was scarce, but where it could be detected, it was either co-expressed with ZO-1 or in direct proximity to ZO-1-positive membrane areas. E-Cad expression was differently distributed over the cell layer. Some cells showed intracellular E-Cad accumulations and a weak membrane staining. In contrast, other cells were intracellularly clear and displayed a strong membrane expression. The partially high amount of intracellular E-Cad suggests an incomplete cellular distribution of E-Cad towards the lateral membrane domain for some of the hepatocytes. For OATP1B1, multiple cells showed a diffuse cellular staining as expected in a correctly polarized hepatocyte. Additionally, we observed membrane areas showing a strong OATP1B1 expression, partly in co-expression with E-Cad or in direct proximity to it. Thus, the majority of hepatocytes cultured between two layers of 100C were well able to form defined, polarized membrane domains.

### PHH SCs between two layers of 90 % collagen and 10 % Matrigel

Morphologically, the cell layers of the 90C/10M cultures resembled very much those of the pure collagen type I cultures. Also, MRP2 and ZO-1 expressions appeared quite similar to those in pure collagen type I. MRP2 accumulated in spots or spotted lines, although in general, MRP2-positive areas were rather few. E-Cad-positive cell-cell contacts were defined more precisely and intracellular accumulations appeared less than in 100C. In contrast, OATP1B1 expression between cells was increased and more comparable to 100M cultures. Taken together, the 90C/10M cultures majorly reflected the polarization of the 100C cultures but started losing the well-defined basolateral membrane domain.

### PHH SCs between two layers of 100 % Matrigel

The PHHs in 100M SCs formed either dense layers like in the collagen-containing cultures or spheroid-like clusters of different sizes. Independent of the cellular arrangement, ZO-1 expression was observed in dense lines and spots between adjacent cells. MRP2 expression however, was rather diffuse with only few spots or lines in co-expression with ZO-1. E-Cad expression was more diffuse, than in the 90C/10M and 100C cultures; accumulations at cell borders could hardly be detected. In contrast to the collagen-containing cultures, OATP1B1 expression traced the whole membrane area of many of the PHHs suggesting an overlap of the basolateral membrane domain with the apical/lateral membrane domain. Consequently, the typical hepatocytic polarization was not achieved.

### Culturing PHHs in sandwich-position between two Matrigel layers favors bile canaliculi formation

**Figure 6:**
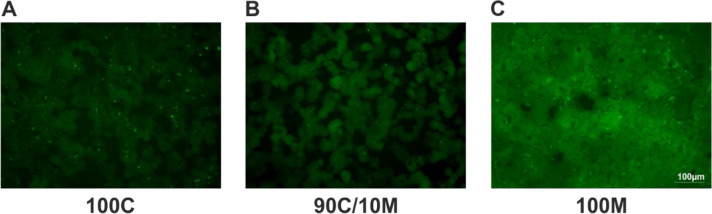
Formation of bile canaliculi in primary human hepatocyte (PHH) sandwich cultures (SCs) between different extracellular matrices (ECMs). PHHs were cultured for 6 days between two layers of (A) pure collagen type I (100C), (B) collagen type I supplemented with 10 % Matrigel (90C/10M) or (C) pure Matrigel (100M). Bile canaliculi were visualized by the fluorescent CDF (5- (and 6) -carboxy-2-, 7-dichlorfluorescein) that is transported into the bile canaliculi via the apical hepatocyte membrane transporter MRP2 (multidrug resistance-associated protein 2). Imaging analysis was performed by three different investigators independently. Representative fluorescence microscopy images are shown. Magnification: 200x. Scale Bar 100 µm. A comprehensive presentation of images from all three donors can be found in Supplementary Figure 7.

The formation of functional bile canaliculi between neighboring PHHs in SCs between 100 % collagen or Matrigel or a mix of 90 % collagen and 10 % Matrigel was assessed by the CDF-assay (Figure 6A-C(Fig. 6)). The fluorescent CDF is secreted into the bile via the apical hepatocyte membrane transporter MRP2 allowing the visualization of the bile canaliculi by fluorescence microscopy. After 6 days of culture, formation of functional bile canaliculi could be observed under all 3 culturing conditions. But while the 100C and 90C/10M cultures showed rather similar pictures of CDF spots and short lines, the bile canaliculi formed by 100M-cultured hepatocytes were longer and more branched. Some cell clusters even formed whole canaliculi networks.

See also the Supplementary data.

## Discussion

The liver's ECM is much more complex than the most widely applied matrices for hepatocyte culture, namely collagen type I and Matrigel. While collagen type I is present all over the liver lobule, there is an abundance of other ECM elements, that are differently distributed over the sinusoid (Martinez-Hernandeza and Amenta, 1993[26]). Although the sinusoid does not exhibit a proper basal lamina, the basement membrane extract Matrigel contains several of these hepatic ECM components, like collagen type IV, laminin and heparan sulfate proteoglycane (Hughes et al., 2010[19]). Our hypothesis was that a combination of the two traditional ECM coatings could prevent the known dedifferentiation of hepatocytes *in vitro*. Therefore, we conducted a thorough and systematic analysis of the influence of different percentages of Matrigel added to collagen type I on the maintenance of PHH differentiation and their functionality within the cellular arrangement.

Our results show that the general metabolic activity of primary hepatocytes is better sustained on mixed collagen-Matrigel coatings than on pure collagen type I or Matrigel. This could be observed especially in the analyses of mitochondrial activity (XTT assay) and protein concentrations. In particular, pure collagen type I cultures displayed a lower functionality than pure Matrigel or Matrigel-supplemented ECMs. The stable and low release of intracellular hepatic enzymes over the course of the cell culture indicates no negative effect of either ECM components on hepatocyte viability. In this regard, the cell activity data represent rather active cellular regeneration and growth than being an indicator for viability. The decline of cell activities on day 6 independent of the used ECMs suggests a lowering of regenerative events and/or growth rates. Taken together, an influence of ECMs on initial cell stress and adherence is a plausible explanation for describing the observed differences in pure collagen type I cultures. The addition of Matrigel seems to dampen post-isolation stress as indicated by a lower initial hepatic enzyme release. It is known that loss of cell-matrix interactions leads to senescence, triggering apoptotic pathways (Gómez-Lechón et al., 1998[16]). Thus, the saturation of open binding sites for ECM components on freshly isolated hepatocytes can curb activated stress pathways. Hence, hepatocytes on the brink of apoptosis have a better chance to survive. Our results on protein concentrations suggest that Matrigel components in general have a beneficial influence on cell adhesion and growth. While general metabolic functions like glucose and lactate formation stabilized with ongoing culture time ECM-independently, critical hepatic functions like urea synthesis were stabilized only in Matrigel-supplemented cultures. We conclude that a certain concentration of Matrigel components can dampen cell isolation stress, support cellular regeneration and growth as well as stabilize hepatic functions in the subsequent culture. Such properties of Matrigel have already been shown (Fraczek et al., 2013[12]). But we have also detected negative effects of pure Matrigel usage. Our approach of adding Matrigel to collagen type I can boost the positive Matrigel influence while negative effects are minimized. 

A dedifferentiation-associated downregulation of albumin, the liver-enriched transcription factor HNF4A and its downstream targets in the cytochrome P450 enzyme group in primary hepatocyte cultures is well described (Elaut et al., 2006[10]). Except for CK-18 and partly CYP2C9, the expression of all genes measured in our study declined in PHHs cultured on pure collagen type I or Matrigel. Concerning ECM dependency, these results are in line with Borlak et al., who found equal transcript expressions of albumin and HNF4A in rat hepatocytes cultured in sandwich configurations on either collagen or Matrigel (Borlak et al., 2015[6]). Concerning time-dependency of gene expression, another group described a recovery of initially downregulated albumin and CYP transcription in PHH MCs on collagen I for 5 days, suggesting an adaptation of the hepatocytes to the culture conditions (Kiamehr et al., 2019[23]). Such an effect was not visible under our culture conditions. The aforementioned study, however, analyzed PHHs that were derived from only one single donor, thus inter-study comparability may be low. In contrast to the downregulated expressions on pure ECMs, albumin mRNA levels were stabilized over the culture time on mixed matrices. Such a clear stabilizing effect of the mixed matrices was not seen for HNF4A, although its mRNA levels on day 6 were higher on most mixed matrix combinations than on pure collagen type I and in part also higher than on pure Matrigel. Our results on pure collagen type I are in line with Wang et al., who also reported diverging effects of collagen I on albumin and HNF4A expressions in a hepatic cell line (Wang et al., 2016[44]). Though albumin and HNF4A are both very early markers of hepatocyte differentiation (Si-Tayeb et al., 2010[37]), our results indicate that their response to ECM-mediated signaling is differentially regulated. Taken together with our functional data, the ECM composition has a clear influence on the maintenance of hepatocyte differentiation *in vitro* and mixed collagen-Matrigel matrices provide a beneficial environment in this regard. Therefore, PHHs were further cultured on collagen supplemented with 10 % Matrigel in a sandwich configuration to investigate the influence of the mixed matrix approach on the ability of hepatocytes to develop a properly polarized phenotype. The concentration of 90 % collagen and 10 % Matrigel was chosen because of its significant stabilization of the polarization marker E-Cad.

In our SC assays, viability of PHHs cultured on 100 % Matrigel was markedly diminished. These observations are in line with investigations on rat hepatocytes cultured on soft polyacrylamide hydrogels linked to either Matrigel or collagen type I (Serna-Márquez et al., 2020[35]). Our results match clinical observations regarding liver cirrhosis, where the deposition of basement membrane components in the sinusoids is accompanied by a decreased hepatic function (Ma and Mei, 2017[25]). We furthermore witnessed a high confluency in our collagen-containing SCs, while the conformation of PHHs cultured between two 100 % Matrigel layers varied between small cell clusters over larger spheroid-like formations to confluent layers. These multicellular arrangements were confirmed in our Phalloidin/Hoechst stainings (Supplementary Figure 6). Analogously diverging cellular arrangements on collagen type I or Matrigel were already reported in Deharde et al. (2016[8]) and by others (Duval et al., 2017[9]; Hamilton et al., 2001[17]; Moghe et al., 1996[28]; Serna-Márquez et al., 2020[35]). In the light of these observations, our results on E-cadherin expression are intriguing. Concerning mRNA expression levels of E-Cad, mixed matrices showed superiority over the pure ECMs and especially over the 100 % Matrigel cultures. Correlating with the transcript data, the immunofluorescence images of PHHs cultured in 100 % Matrigel SCs revealed a diffuse intracellular E-cadherin signaling with hardly any expression at cell borders. Moghe et al. already hinted at an altered E-cadherin expression in Matrigel-based hepatocyte cultures a while ago (Moghe et al., 1996[28]). E-cadherin is a key element of adherens junctions, thereby mediating cell-cell contacts and outside-in signaling. It further participates in the organization of tight junctions, thus influencing polarization through the determination of apical and basolateral membrane areas (Treyer and Müsch, 2013[40]). In accordance with our former observations (Deharde et al., 2016[8]), the tight junction and apical domain markers ZO-1 and MRP2 were expressed diffusely in the pure Matrigel SCs. Additionally, OATP1B1 traced whole membrane areas, pointing to an incomplete repolarization. The expression patterns of polarization markers in 90C/10M were rather similar to 100C. Though, the pure collagen type I SCs displayed a better locally organized OATP1B1 signaling. The expression of the drug membrane transporter OATP1B1 that is selectively found in the sinusoidal hepatocyte membrane, declines rapidly in hepatocyte cultures (Ulvestad et al., 2011[41]). While a comparative study did not observe differences in OATP1B1 mRNA or protein expression after 5 culture days in collagen or Matrigel SCs (Schaefer et al., 2012[34]), our observations hint at a negative influence of an increasing Matrigel content on its maintenance. 

Although the polarization marker pattern in the 100M SCs did not appear well organized, the accumulation of actin filaments around bile canaliculi and the CDF assay revealed the formation of functioning bile canaliculi in the 100M SCs building even more complex formations than in the collagen-containing SCs. Successful development of an apical-basolateral polarity was demonstrated in a HepG2 cell line that harbors a defect in E-cadherin localization leading to its intracellular retainment comparable to our observations in the 100M SCs (Théard et al., 2007[39]). Development of a hepatocytic polarization and formation of functional bile canaliculi was even feasible in matrix-free HepG2/C3A spheroids (Sharma et al., 2019[36]). Therefore, the mere existence of cell-cell contacts, seems to enable polarization. Whether such canaliculi formed between hepatocytes without a supporting ECM are stable, leaves room for speculations. In our previous study, the bile canaliculi formed by PHHs in pure Matrigel SCs showed the least stability (Deharde et al., 2016[8]). Moreover, hepatocytes are able to secrete ECM components which have been shown to play a role in cellular remodeling in hepatic spheroids (Michielin et al., 2020[27]; Wells, 2008[46]). Apart from that, a novel single-cell culture approach was described, in which hepatocytes were able to form hemi-canaliculi and displayed polarization independently of cell-cell-contacts, only triggered by the ECM and immobilized cadherin (Zhang et al., 2020[50]). While in the presence of cadherin, the nature of the underlying ECM (Matrigel, laminin or collagen) did not influence the induction of polarity, hepatocyte attachment failed on Matrigel at low cadherin densities, again suggesting a crucial role for cadherin in hepatocyte polarity induction. This attribution is confirmed by the observed downregulation of E-cadherin in hepatocellular carcinoma (HCC). The concomitant loss of cell adhesions and polarization has been linked to epithelial-mesenchymal transition (EMT) and the acquisition of invasive behavior (Lu et al., 2018[24]). Laminin, the major component of Matrigel, was also linked to EMT (Giannelli et al., 2005[13]). As laminin is mainly deposited in the periportal area of the liver sinusoid, where immature hepatocytes proliferate and has been shown to be upregulated in HCC tissues, it is associated with stemness and dedifferentiation (Giannelli et al., 2016[14]; Mak and Mei, 2017[25]). We conclude, that although the PHHs cultured on pure Matrigel showed several features of dedifferentiation and especially an altered E-cadherin expression, Matrigel offers a framework for the formation of cell-cell contacts that enable hepatocyte polarization. In addition to cell-cell contacts, cell-matrix interactions are crucial for the maintenance of a differentiated hepatocyte phenotype. We suggest that mixed collagen-Matrigel matrices combine the positive traits of both ECM types and allow for a better development of cell-cell as well as cell-matrix contacts.

A critical issue in the use of Matrigel is that it contains growth factors, like e.g. transforming growth factor β (TGF-β), epidermal growth factor (EGF), insulin-like growth factor 1, bovine fibroblast growth factor (bFGF), and platelet-derived growth factor (PDGF) (Vukicevic et al., 1992[43]). Considering the systematic approach of our study with application of many ascending percentages of Matrigel in our ECM compositions, we did not utilize growth factor-reduced Matrigel for economic reasons. With regard to the positive effects we could observe in PHHs cultured on ECMs containing Matrigel, this arises the question of a possible confounding effect due to the presence of growth factors. It is known, that EGF, FGF and PDGF play a major role in liver regeneration and therefore are closely linked to dedifferentiation events during hepatocyte proliferation (Hoffmann et al., 2020[18]). In contrast, TGF-β activates the epithelial to mesenchymal transition (EMT) in hepatocytes leading also to dedifferentiation and loss of function (Xu et al., 2009[47]). Taken together, growth factors are linked to a deteriorating effect on hepatic differentiation. It was shown that the ECM of Matrigel maintained the hepatic differentiation and rendered them unresponsive to growth factor responsiveness, shown by a decrease of multiple proliferation promoting proto-oncogenes (Elaut et al., 2006[10]). Therefore, an influence of the growth factors on the results is very unlikely. However, future studies, seeking to validate our findings, should take a growth factor interference into account and utilize growth factor-reduced Matrigel.

Taken together, our study gives insights into ECM-dependent metabolism and polarity-associated functions of cultured PHHs on different levels. We provide a comprehensive comparison of the application of the two popular ECMs collagen type I and Matrigel and their combinations in monolayer and SCs over the course of 6 days. Our results strongly hint at ECM-dependency of the maintenance of the differentiated hepatocytic phenotype, while polarization is tied to the ability to form cell-cell contacts. Notwithstanding, a successful establishment of an *in vivo*-like *in vitro* liver model relies on both traits. Mixed collagen-Matrigel coatings showed superior properties concerning general hepatic metabolic functions. Nonetheless, Matrigel supplementation also led to a concentration-dependent loss of basolateral polarization. Therefore, the Matrigel supplementation has to be carefully adapted to gain optimum results. Preclinical assessments of drug-interactions in *in vitro* liver models rely on both biliary excretion and a physiological drug uptake via basolateral membrane transporters (Barton et al., 2013[3]). Therefore, further investigations on the functional level, e.g. on drug uptake and excretion activity of sandwich-cultured PHHs on mixed matrices are required to further validate this otherwise simple and cost-effective approach for the harmonization of the differentiated PHH phenotype *in vitro*.

## Declaration

### Conflict of interest

The authors declare no conflict of interest. The funders had no role in the design of the study; in the collection, analyses, or interpretation of data; in the writing of the manuscript, or in the decision to publish the results.

### Acknowledgments

We cordially thank Clara Paula Lippold and Julian Connor Eckel at SIKT Leipzig for their support and their contributions to preliminary investigations and the team from Leipzig University Medical Center for the fruitful collaboration. We further want to express our gratitude to Dr. Norbert Köhler from the Institute for Medical Informatics, Statistics and Epidemiology at Leipzig University for his advice and assistance with the statistical analyses. The study was supported by the Federal Ministry of Education and Research (BMBF, Germany) within the research network Liver Systems Medicine (LiSyM) Krebs [grant number: BMBF 031L0256C] and partly funded with tax money approved by the delegates of the Saxon state parliament. L.S. receives funding as a participant in the clinician scientist program of Leipzig University Medicine.

## Supplementary Material

Supplementary information

Supplementary data

## Figures and Tables

**Table 1:**
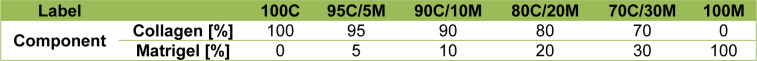
Mixing ratios of collagen type I and Matrigel for monolayer cultivation

**Table 2:**
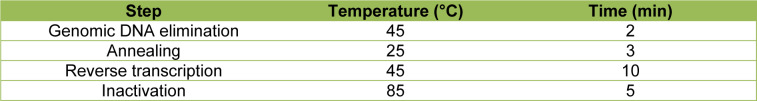
Thermocycling conditions of the reverse transcription reaction

**Table 3:**
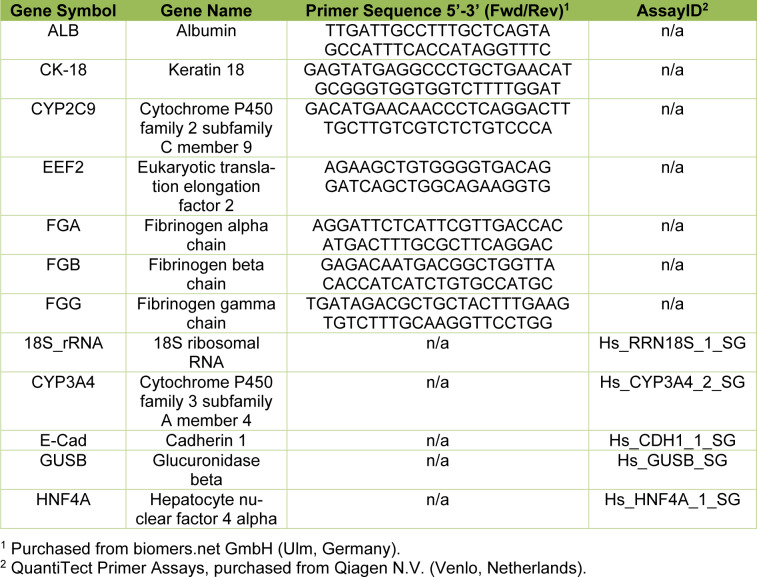
Primers utilized for RT-qPCR

**Table 4:**
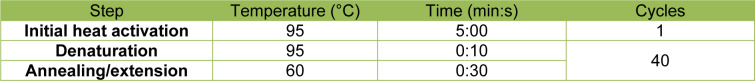
Thermocycling profile for qPCR reactions

**Table 5:**
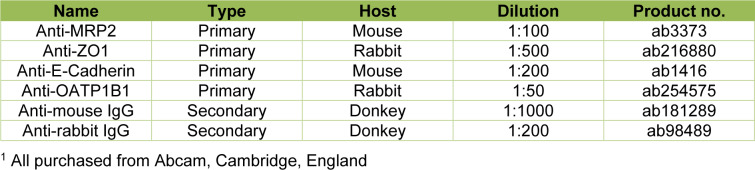
Antibodies used for immunofluorescence staining^1^
