# Supplementary material for: Optimization of extracellular matrix for primary human hepatocyte cultures using mixed collagen-Matrigel matrices
Source: EXCLI J. 2023 Jan 4;22:12–34. doi: 10.17179/excli2022-5459 (PMC9837384; doi:10.17179/excli2022-5459)
Supplement: Supplementary information [file EXCLI-22-12-s-001.pdf]

EXCLI Journal 2023;22:12-34 – ISSN 1611-2156
Received: September 22, 2022, accepted: November 17, 2022, published: January 04, 2023

Supplementary information to:

Original article:

OPTIMIZATION OF EXTRACELLULAR MATRIX FOR PRIMARY HUMAN HEPATOCYTE CULTURES USING MIXED COLLAGEN-MATRIGEL MATRICES

Lena Seidemann[1,2], Sarah Prinz[1,2], Jan-Constantin Scherbel[1,2], Christina Götz[1,2], Daniel Seehofer[1,2], Georg Damm[1,2,*]

[1] Department of Hepatobiliary Surgery and Visceral Transplantation, University Hospital, Leipzig University, Liebigstr. 20, 04103 Leipzig, Germany
[2] Saxonian Incubator for Clinical Translation (SIKT), Leipzig University, Philipp-Rosenthal-Str. 55, 04103 Leipzig, Germany

[*] **Corresponding author:** Georg Damm, Department of Hepatobiliary Surgery and Visceral Transplantation, University Hospital, Leipzig University, Liebigstr. 20, 04103 Leipzig, Germany. Tel.: +49-341-9739656, E-mail: georg.damm@medizin.uni-leipzig.de

https://dx.doi.org/10.17179/excli2022-5459

This is an Open Access article distributed under the terms of the Creative Commons Attribution License (http://creativecommons.org/licenses/by/4.0/).

EXCLI Journal 2023;22:12-34 – ISSN 1611-2156
Received: September 22, 2022, accepted: November 17, 2022, published: January 04, 2023

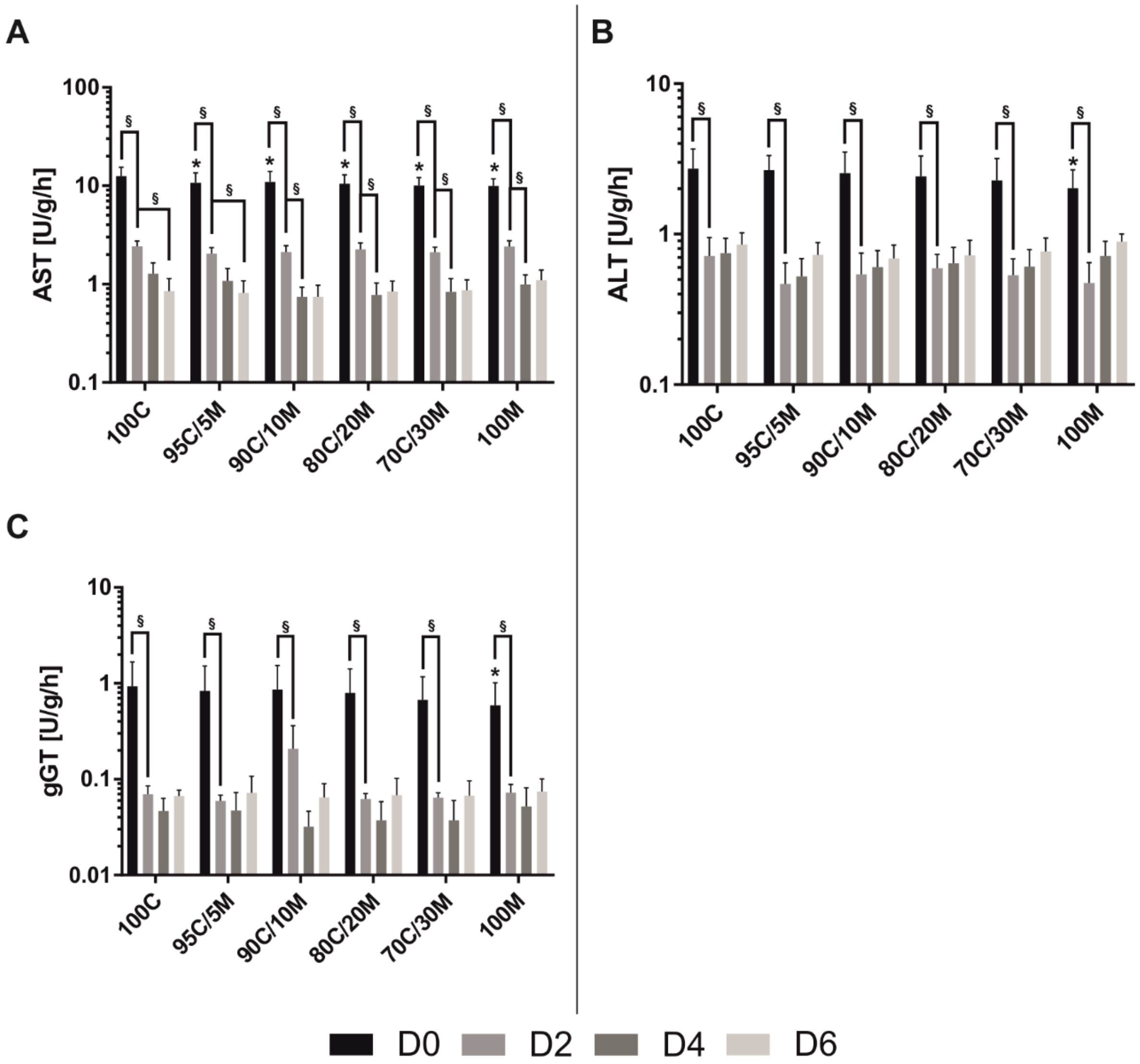

Supplementary Figure 1: Liver enzyme activities in supernatants of primary human hepatocytes (PHHs) in monolayer cultures (MCs) on different extracellular matrices (ECMs). PHHs were cultured for up to 6 days on pure collagen type I (100C), on ECMs based on collagen type I (C), supplemented with ascending percentages of Matrigel (M): 95C/5M, 90C/10M, 80C/20M, 70C/30M or on pure Matrigel (100M). Enzyme activities of (**A**) AST (aspartate aminotransferase); (**B**) ALT (alanine aminotransferase) and (**C**) gGT (gamma-glutamyl transferase) were measured in cell culture supernatants and normalized to the protein concentration and duration of the cell culture until supernatant collection. Data are shown as the mean + SEM, n = 5, two-way ANOVA and Bonferroni correction for multiple testing, statistical significance was assumed at $p \leq 0.0332$. Significant differences between PHHs cultured on one of the various matrices in comparison to PHHs cultured on 100C of the same day are marked with *. Significant differences between PHHs cultured on one of the various matrices in comparison to PHHs cultured on 100M of the same day are marked with #. Significant differences observed at different time points of PHHs cultured on the same ECM are marked with §. Selected comparisons are shown; for details on the statistical evaluation, see Supplementary Table 1D, supplementary data.

EXCLI Journal 2023;22:12-34 – ISSN 1611-2156
Received: September 22, 2022, accepted: November 17, 2022, published: January 04, 2023

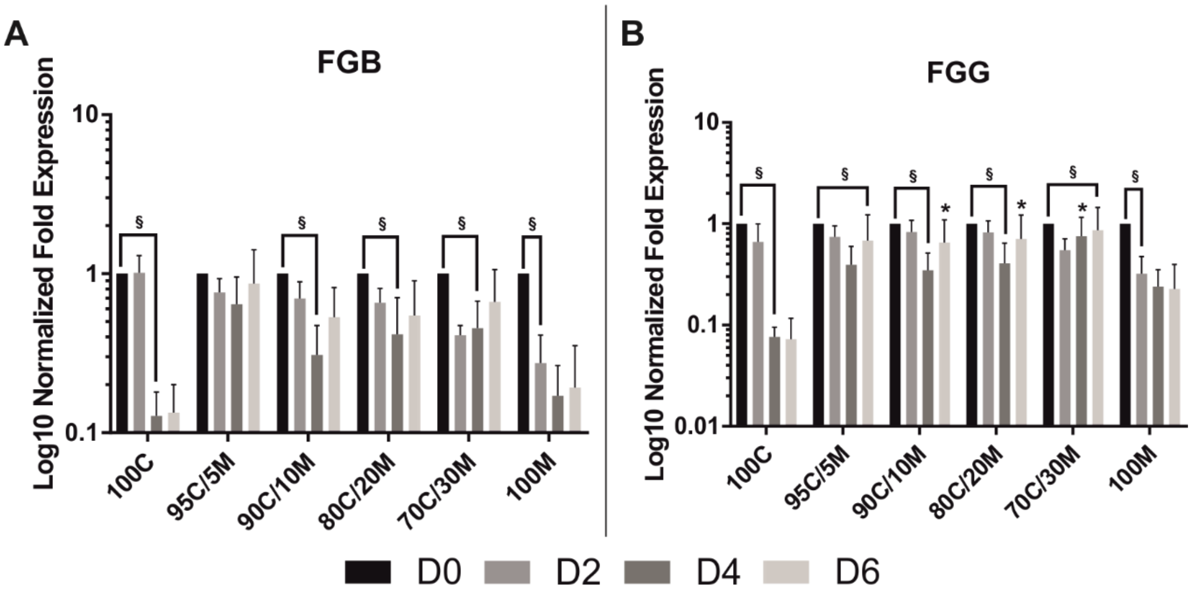

Supplementary Figure 2: Gene expression of fibrinogen beta chain (FGB) and fibrinogen gamma chain (FGG) in primary human hepatocytes (PHHs) in monolayer cultures (MCs) on different extracellular matrices (ECMs). PHHs were cultured for up to 6 days on pure collagen type I (100C), on ECMs based on collagen type I (C), supplemented with ascending percentages of Matrigel (M): 95C/5M, 90C/10M, 80C/20M, 70C/30M or on pure Matrigel (100M). Messenger RNA levels of (**A**) FGB and (**B**) FGG were determined by RT-qPCR. Fold gene expressions were normalized to day 0 of each cultivation type. Data are shown as the mean of normalized fold gene expressions + SEM on a log10-scale, n = 3, two-way ANOVA and Bonferroni correction for multiple testing, statistics were performed on ΔCT values, statistical significance was assumed at $p \leq 0.0332$. Significant differences between PHHs cultured on one of the various matrices in comparison to PHHs cultured on 100C of the same day are marked with *. Significant differences between PHHs cultured on one of the various matrices in comparison to PHHs cultured on 100M of the same day are marked with #. Significant differences observed at different time points of PHHs cultured on the same ECM are marked with §. Selected comparisons are shown; for details on the statistical evaluation, see Supplementary Table 1E, supplementary data.

EXCLI Journal 2023;22:12-34 – ISSN 1611-2156
Received: September 22, 2022, accepted: November 17, 2022, published: January 04, 2023

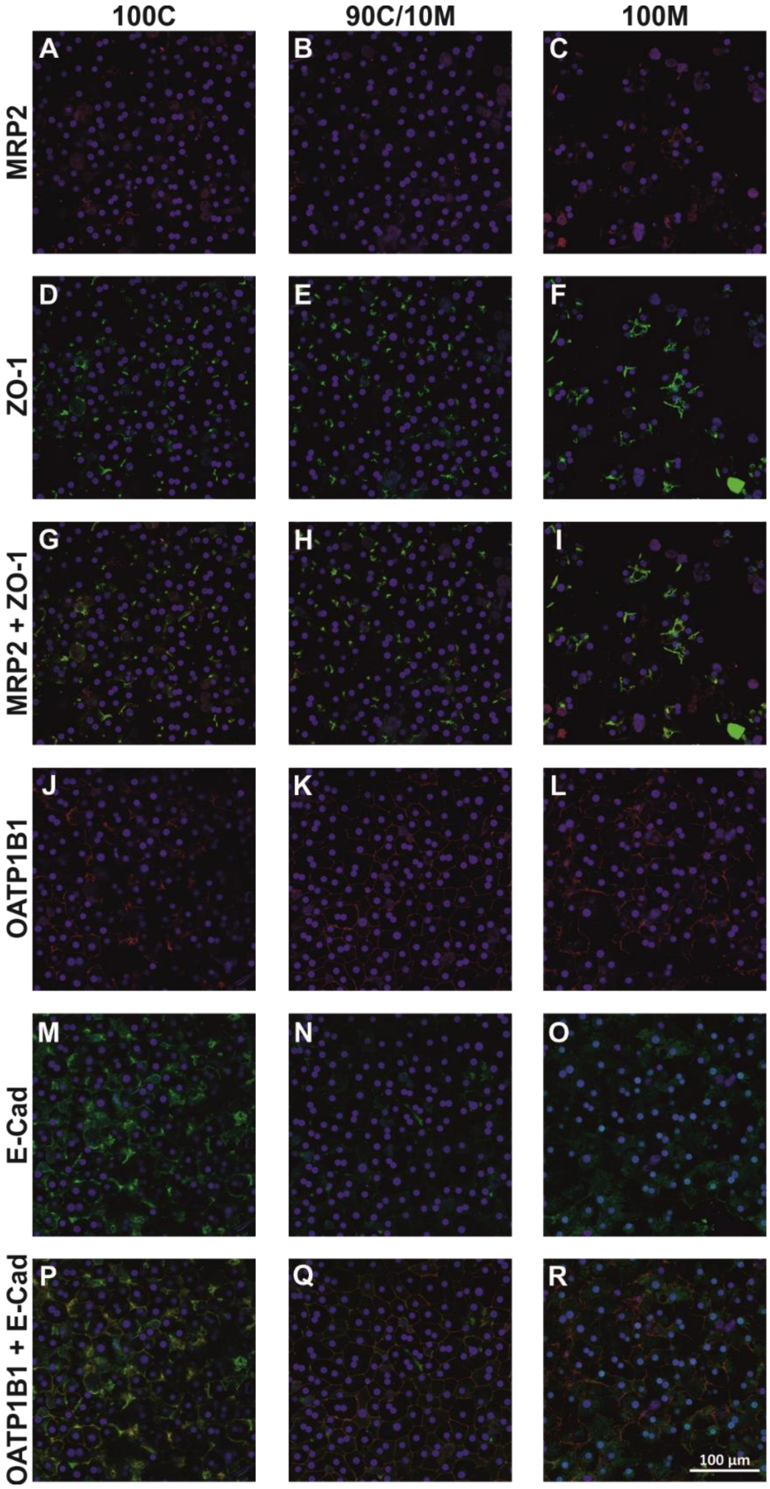

Supplementary Figure 3: Investigation of primary human hepatocyte (PHH) polarization in sandwich cultures (SCs) between different extracellular matrices (ECMs). PHHs from donor 1 were cultured for 6 days between two layers of pure collagen type I (100C; first column), collagen type I supplemented with 10 % Matrigel (90C/10M; second column) or pure Matrigel (100M; third column). The expression of the apical membrane-associated MRP2 (multidrug resistance-associated protein 2; **A-C**; red) and the tight junction protein ZO-1 (zonula occludens-1; **D-F**; green) are displayed individually and merged (**G-I**). Also, the expression of the basal membrane-associated OATP1B1 (organic anion transporting polypeptide 1B1; **J-L**; red) and the adherens junction-associated protein E-cadherin (**M-O**; green) are displayed individually and merged (P-R). Cell nuclei were stained with Hoechst (blue). Imaging analysis was performed by three different investigators independently. Representative fluorescence microscopy images are shown. Magnification: 200x. Scale Bar 100 µm.

EXCLI Journal 2023;22:12-34 – ISSN 1611-2156
Received: September 22, 2022, accepted: November 17, 2022, published: January 04, 2023

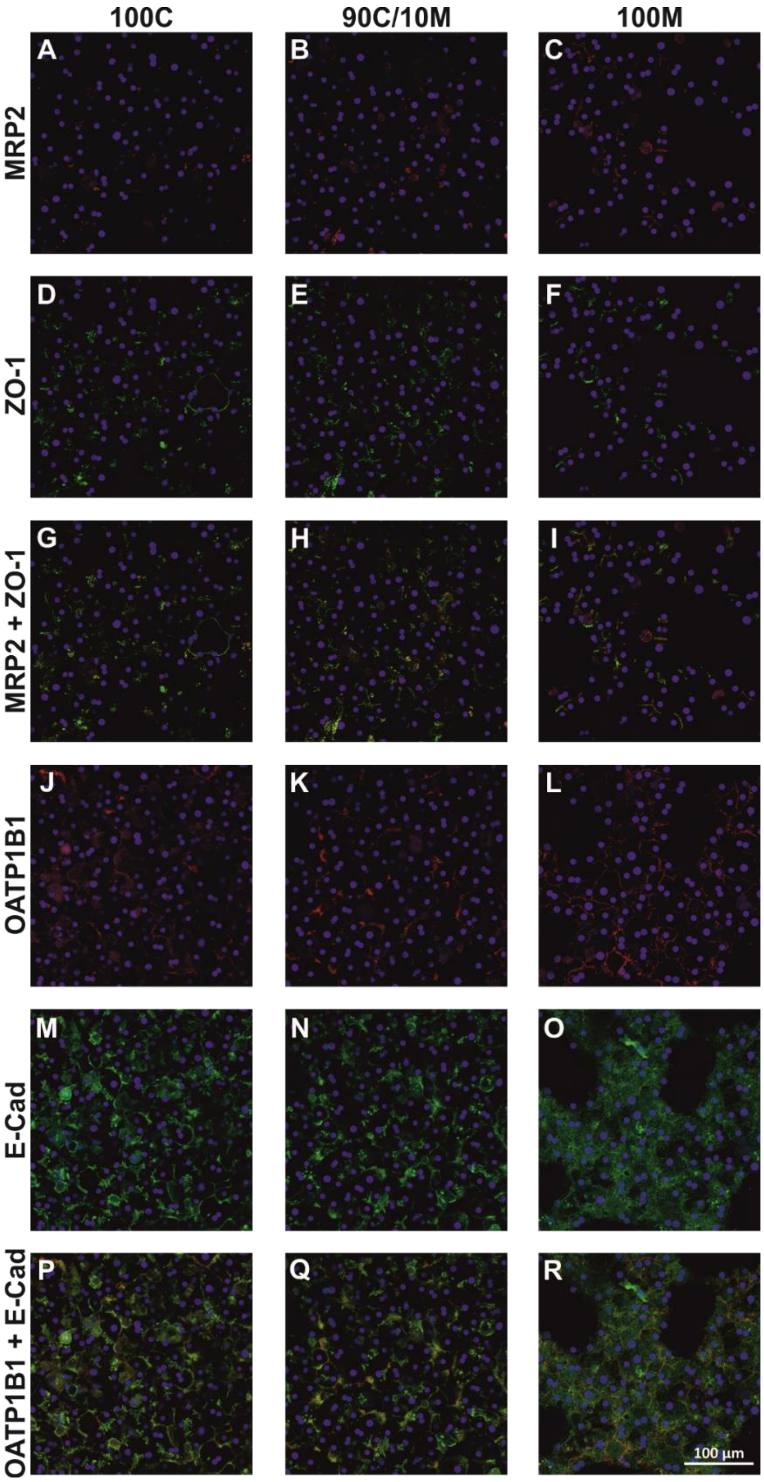

Supplementary Figure 4: Investigation of primary human hepatocyte (PHH) polarization in sandwich cultures (SCs) between different extracellular matrices (ECMs). PHHs from donor 2 were cultured for 6 days between two layers of pure collagen type I (100C; first column), collagen type I supplemented with 10 % Matrigel (90C/10M; second column) or pure Matrigel (100M; third column). The expression of the apical membrane-associated MRP2 (multidrug resistance-associated protein 2; **A-C**; red) and the tight junction protein ZO-1 (zonula occludens-1; **D-F**; green) are displayed individually and merged (**G-I**). Also, the expression of the basal membrane-associated OATP1B1 (organic anion transporting polypeptide 1B1; **J-L**; red) and the adherens junction-associated protein E-cadherin (**M-O**; green) are displayed individually and merged (**P-R**). Cell nuclei were stained with Hoechst (blue). Imaging analysis was performed by three different investigators independently. Representative fluorescence microscopy images are shown. Magnification: 200x. Scale Bar 100 µm.

EXCLI Journal 2023;22:12-34 – ISSN 1611-2156
Received: September 22, 2022, accepted: November 17, 2022, published: January 04, 2023

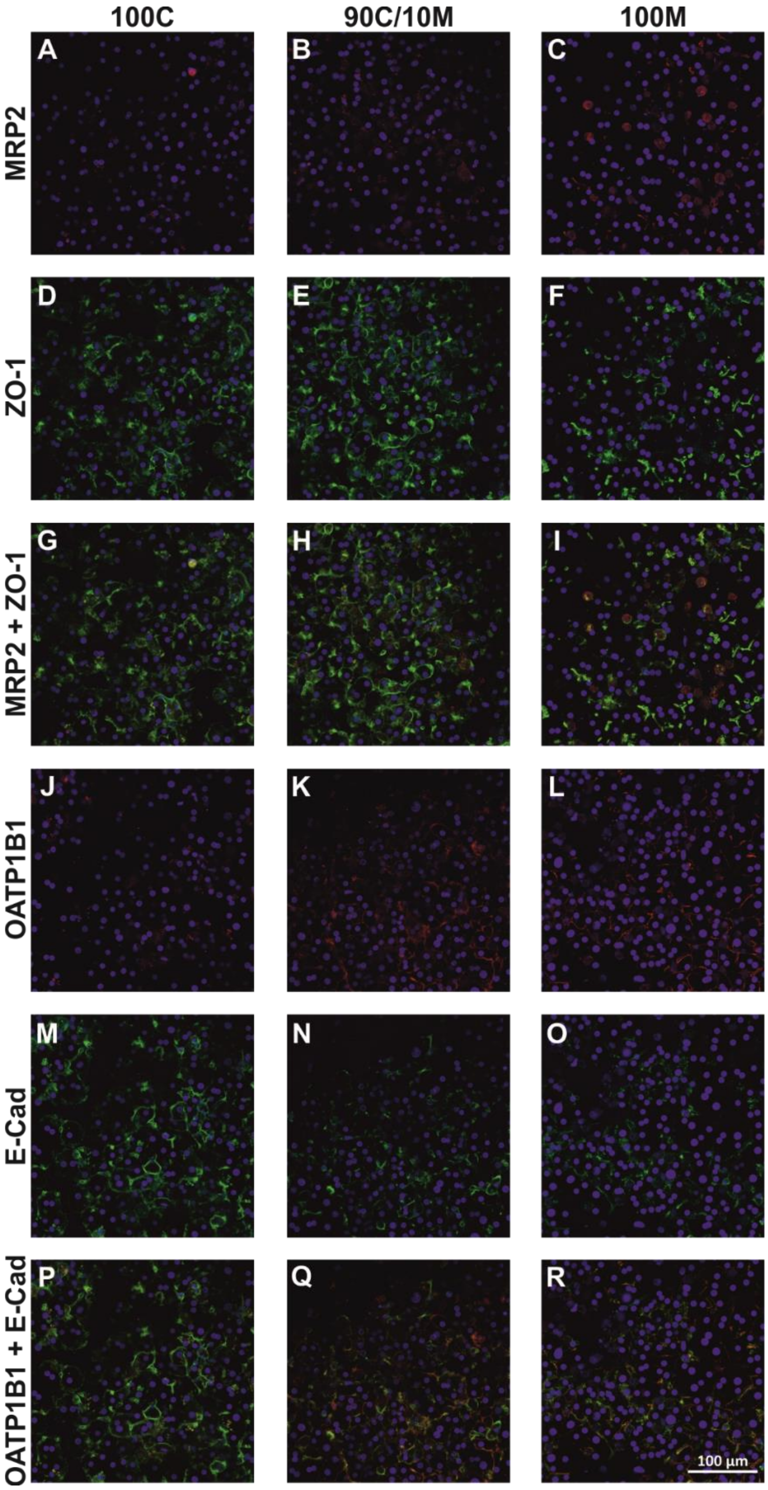

Supplementary Figure 5: Investigation of primary human hepatocyte (PHH) polarization in sandwich cultures (SCs) between different extracellular matrices (ECMs). PHHs from donor 3 were cultured for 6 days between two layers of pure collagen type I (100C; first column), collagen type I supplemented with 10 % Matrigel (90C/10M; second column) or pure Matrigel (100M; third column). The expression of the apical membrane-associated MRP2 (multidrug resistance-associated protein 2; **A-C**; red) and the tight junction protein ZO-1 (zonula occludens-1; **D-F**; green) are displayed individually and merged (**G-I**). Also, the expression of the basal membrane-associated OATP1B1 (organic anion transporting polypeptide 1B1; **J-L**; red) and the adherens junction-associated protein E-cadherin (**M-O**; green) are displayed individually and merged (**P-R**). Cell nuclei were stained with Hoechst (blue). Imaging analysis was performed by three different investigators independently. Representative fluorescence microscopy images are shown. Magnification: 200x. Scale Bar 100 µm.

EXCLI Journal 2023;22:12-34 – ISSN 1611-2156
Received: September 22, 2022, accepted: November 17, 2022, published: January 04, 2023

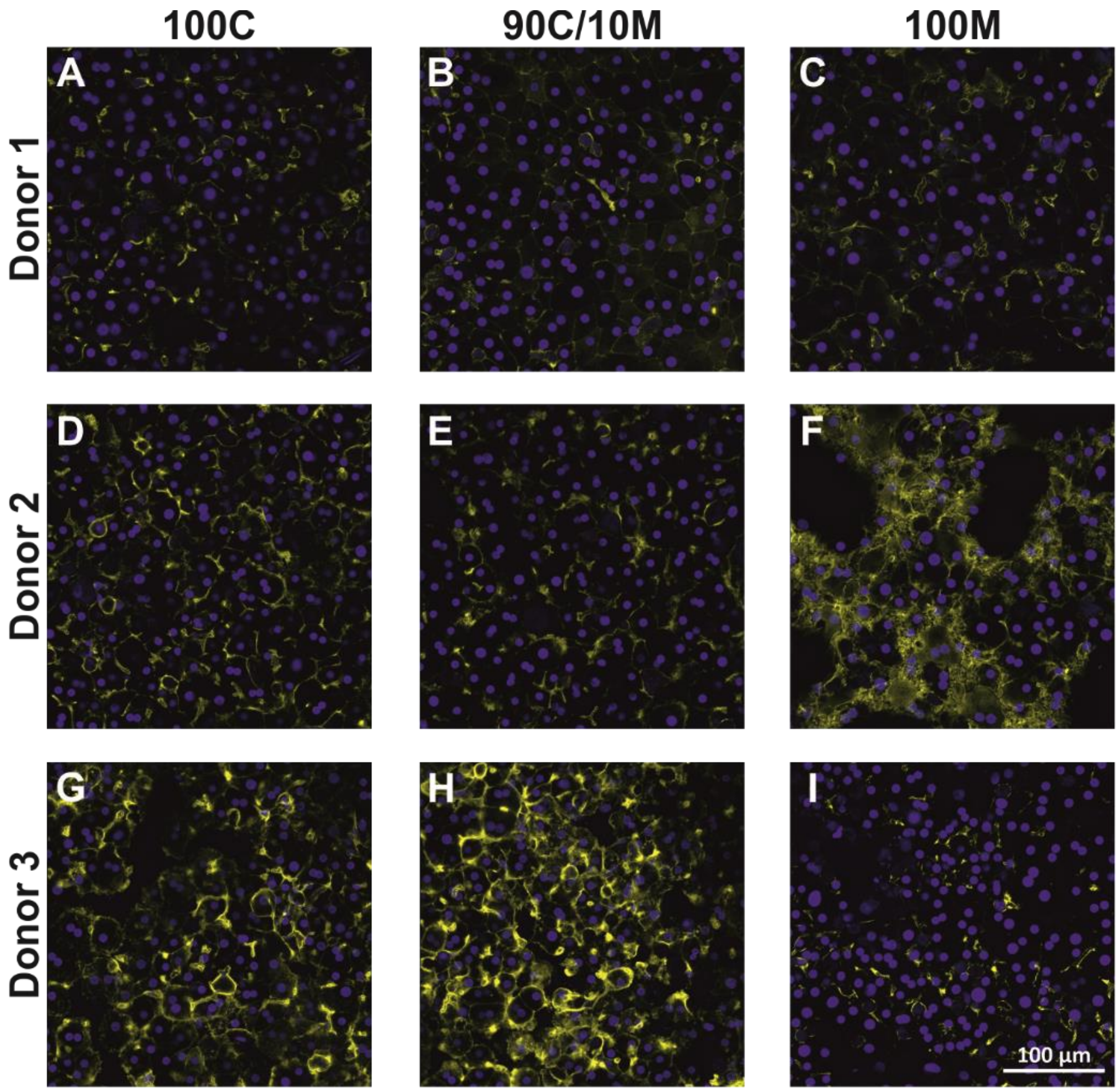

Supplementary Figure 6: Investigation of primary human hepatocyte (PHH) morphology and multicellular arrangement in sandwich cultures (SCs) between different extracellular matrices (ECMs). PHHs from 3 donors (donor 1: **A-C**; donor 2: **D-F**; donor 3: **G-I**) were cultured for 6 days between two layers of pure collagen type I (100C; first column), collagen type I supplemented with 10 % Matrigel (90C/10M; second column) or pure Matrigel (100M; third column). The actin cytoskeleton was stained with phalloidin (yellow). Cell nuclei were stained with Hoechst (blue). Imaging analysis was performed by three different investigators independently. Representative fluorescence microscopy images are shown. Magnification: 200x. Scale Bar 100 µm.

EXCLI Journal 2023;22:12-34 – ISSN 1611-2156
Received: September 22, 2022, accepted: November 17, 2022, published: January 04, 2023

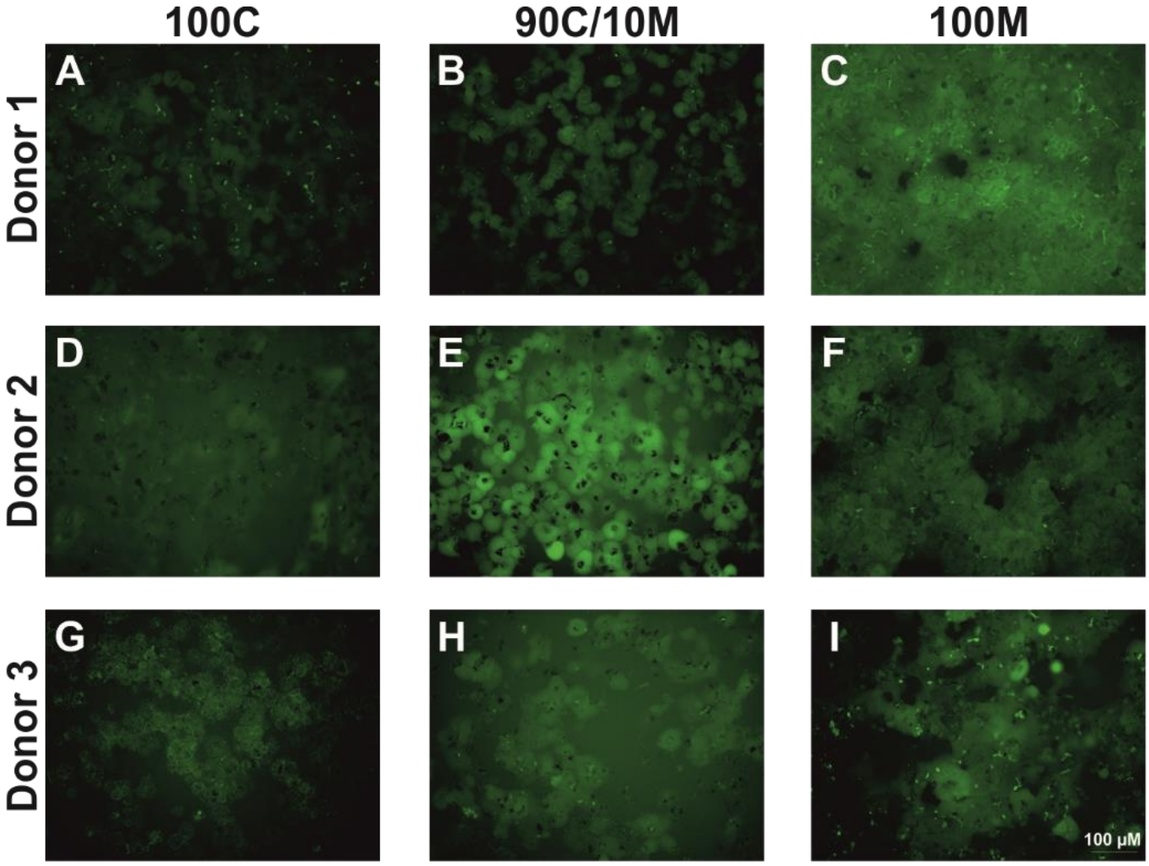

Supplementary Figure 7: Formation of bile canaliculi in primary human hepatocyte (PHH) sandwich cultures (SCs) between different extracellular matrices (ECMs). PHHs from 3 donors (donor 1: **A-C**; donor 2: **D-F**; donor 3: **G-I**) were cultured for 6 days between two layers of pure collagen type I (100C; first column), collagen type I supplemented with 10 % Matrigel (90C/10M; second column) or pure Matrigel (100M; third column). Bile canaliculi are visualized by the fluorescent CDF (5- (and 6) -carboxy-2-, 7-dichlorfluorescein) that is transported into the bile canaliculi via the apical hepatocyte membrane transporter MRP2 (multidrug resistance-associated protein 2). Imaging analysis was performed by three different investigators independently. Representative fluorescence microscopy images are shown. Magnification: 200x. Scale Bar 100 µm.
